# From Evidence-Based Medicine to Precision Health: Using Data to Personalize Care

**DOI:** 10.5935/abc.20180240

**Published:** 2018-12

**Authors:** Marcio Sommer Bittencourt

**Affiliations:** 1 Centro de Medicina Preventiva do Hospital Israelita Albert Einstein e Faculdade de Medicina da Faculdade Israelita de Ciência da Saúde Albert Einstein, São Paulo - Brazil; 2 Centro de Pesquisa Clínica e Epidemiológica - Hospital Universitário e Instituto do Câncer do Estado de São Paulo - Universidade de São Paulo, São Paulo, SP - Brazil; 3 Diagnósticos da América (DASA), São Paulo, SP - Brazil

**Keywords:** Evidence Based Medicine/methods, Precision Medicine/trends, Comprehensive Health Care, Models,Educational, Teaching/methods, Research

The historical practice of medicine has evolved over centuries based on empirical knowledge derived from experience and observation rather than rigorous scientific data. Over the second half of the twentieth century, this form of medical knowledge progress was largely supplanted by rigorous scientific data collection, particularly in the realm of cardiovascular diseases, where virtually every new drug discovery has been thoroughly evaluated in randomized clinical trials (RCT). The impressive improvement in the quality of the information provided by such study design has led to the development of an entirely new field in the medical knowledge which became known as Evidence-Based Medicine (EBM).^1^

EBM soughs to move away from empirical information by providing a structured grading of the epistemological strength of evidence available. It further requires the highest levels of evidence to give strong recommendations for or against the use of any particular therapy. By this approach, RCTs are considered among the highest quality study designs which support those stronger recommendations. Still, despite their capability to avoid confounding and other biases, RCTs conclusions can only be interpreted as an overall averaged benefit for the collective population included in the study. Although such information may suffice to document the effect of any given therapy at the population level, this does not necessarily apply to any individual patient. While some individuals may benefit considerably more than the average population included in the study, other might benefit significantly less, whereas no benefit or even significant harm may occur in some individuals.

Moreover, the external validity in other subgroups of individuals is even more challenging. Although a significant proportion of drugs routinely used in medicine and cardiology are only approved to rather strict clinical indications, most clinicians extrapolate evidence beyond the validated population, including many subgroups of individuals in whom the benefit documented in the initial studies is unlikely to be replicated or in individuals whose risk for complications or side effects might be larger than in the initial cohort.

Though much of these limitations have long been known by individuals working with EBM, not too long ago little more than simple subgroup analysis could be performed in the quest to identify individuals more or less likely to benefit from the tested therapy. Since the identification of those individuals with unexpected response to therapy is rather complex, the simple subgroup analysis lacked the nuance needed to sort the wheat from the chaff in most cases.

Over the last decades, the development of two different fields has led medicine to change this paradigm. On one hand, the development of genetics and genomics provided extensive data on the differences between individuals that might, at least partially, explain the individual variability in risk for various diseases, its prognosis, response to therapy or risk for side effects. On the other hand, data science and computational power developed to an extent that allows data processing at orders of magnitude larger than previously known. This improvement in computational power allowed the capability to handle large amounts of data, such as those provided in genetic studies. Therefore, the insights provided by the combined use of those two fields can help tailor individualized treatment. Within this context, the concept of precision and individualized medicine have developed over the last couple of years.^2^

Precision medicine has been defined as a medical model using molecular profiling technologies to improve diagnostic accuracy, prognosis definition and tailor the right therapeutic strategy to the right person at the right time.^3^ However, this definition has a limited scope on the potential of personalized care at the current state of healthcare delivery. First, individualized care now extends towards the broader spectrum of health care including primary and primordial prevention, as well as health promotion. Consequently, the broader term of precision health, not precision medicine may seem more appropriate. Within this concept, one can only naturally understand that to provide the full board of precision health to patients there is a compelling need to extend the data collection beyond genetic, molecular or genomic profiling to incorporate a more "holistic" definition of health. This health profiling should further embrace other social and environmental data but should also include the entirely new field of patient-generated data provided by newer devices such as smartphones, smartwatches and other wearables that can provide vast amounts of continuous monitoring data from each individual through exceedingly long periods of time. Finally, in order to provide truly personalized precision health, each healthcare provider will need to factor in individual patients' preferences.

This entire concept of personalized health is still at its early stages, and the exact blending of those parameters are not yet known. However, with the fast pace of experimentation allowed by studies derived for large datasets of real-life information, one can foresee this becoming routine standard of care in a not too distant future.

## References

[1] (1992). Evidence-based medicine A new approach to teaching the practice
of medicine. JAMA.

[2] Erden A (2015). Personalized medicine. Yale J Biol Med.

[3] Nimmesgern E, Benediktsson I, Norstedt I (2017). Personalized medicine in Europe. Clin Trans Sci.

